# The Psychological Impact of COVID-19 Pandemic on Women’s Mental Health during Pregnancy: A Rapid Evidence Review

**DOI:** 10.3390/ijerph18137112

**Published:** 2021-07-02

**Authors:** Monica Ahmad, Laura Vismara

**Affiliations:** Department of Pedagogy, Psychology, Philosophy, Faculty of Human Studies, University of Cagliari, 09124 Cagliari, Italy; m.ahmad4@studenti.unica.it

**Keywords:** COVID-19, maternal mental health, anxiety, depression, perinatality

## Abstract

Background: The perinatal period is a particularly vulnerable period in women’s lives that implies significant physiological and psychological changes that can place women at higher risk for depression and anxiety symptoms. In addition, the ongoing pandemic of coronavirus disease 2019 (COVID-19) is likely to increase this vulnerability and the prevalence of mental health problems. This review aimed to investigate the existing literature on the psychological impact of the COVID-19 pandemic on women during pregnancy and the first year postpartum. Method: The literature search was conducted using the following databases: Pubmed, Scopus, WOS—web of science, PsycInfo and Google Scholar. Out of the total of 116 initially selected papers, 17 have been included in the final work, according to the inclusion criteria. Results: The reviewed contributions report a moderate to severe impact of the COVID-19 outbreak on the mental health of pregnant women, mainly in the form of a significant increase in depression—up to 58% in Spain—and anxiety symptoms—up to 72% in Canada. In addition to the common psychological symptoms, COVID-19-specific worries emerged with respect to its potential effects on pregnancy and the well-being of the unborn child. Social support and being engaged in regular physical activities appear to be protective factors able to buffer against the effects of the pandemic on maternal mental health. Conclusions: Despite the limitations of the study design, the evidence suggests that it is essential to provide appropriate psychological support to pregnant women during the emergency in order to protect their mental health and to minimize the risks of long-term effects on child development.

## 1. Introduction

On 12 January 2020, the World Health Organization (WHO) officially announced the coronavirus disease 2019 (COVID-19), originating in Wuhan in December 2019, as a pandemic. 

In the course of most infectious disease outbreaks, restrictive measures can be necessary to stop the virus. With the aim of limiting Severe Acute Respiratory Syndrome Coronavirus 2 (SARS-CoV-2) propagation, governments around the world have imposed some restrictions, such us national lockdowns and social distancing. A recent review [1] suggested that restrictive measures are often associated with negative psychological effects that can still be identified months or years later, and highlighted the impact of quarantine and isolation on mental health. 

Indeed, the actual outbreak is leading to psychological distress and increased mental health problems, such as stress, anxiety, depressive symptoms, insomnia, denial, anger and fear [2]. Psychological distress and mood disorders seem most likely in more vulnerable populations [3,4,5], such as pregnant women.

Maternal mental health is particularly important to consider, due to the increased risk for depression and anxiety [6]. Pregnancy and the postpartum period, especially for first time mothers, have been identified as delicate periods in a woman’s life that are accompanied by significant social, psychological and also physiological changes [7,8], and for this reason pregnant women have been considered a high-risk population.

Several studies have reported that the perinatal period is a time characterized by increased risk for emotional disorders such as depression, anxiety, and trauma-related disorders, especially in the presence of stress conditions [8,9,10]. This is also true for pregnant and postpartum women and their infants in the face of emergencies or natural disasters [11,12].

Indeed, during the SARS outbreak, pregnant women may have concerns about their own health and about the health of their unborn babies, and may display fears relating to pregnancy, to childbirth, or both. Additionally, feelings of uncertainty (characteristic of an epidemic) represent a significant stressor that can increase distress in pregnant women [13].

Overall, these complex and multiple variables may affect both mothers and their children’s physical and psychological health, in short-, medium- and long-term periods [14,15,16,17]. Therefore, the condition of the COVID-19 pandemic and associated factors could produce additional stress for women during perinatality and accentuate this predisposition [3,18]. For these reasons and due to the negative effect of psychological distress during pregnancy on the health of mothers and their offspring, priority should be given to support maternal mental health in the perinatal period [19,20]. These issues suggest that research is necessary to explore the effects of the COVID-19 pandemic on women during perinatality. The current review was designed to summarize the existing literature on the psychological impact of the COVID-19 pandemic on pregnant women.

## 2. Material and Methods

This research was conducted as a rapid review. Rapid reviews follow the guidelines for systematic reviews, but are simplified in order to accelerate the process of traditional reviews to produce rapid evidence [21].

### 2.1. Search Strategy

The Pubmed, Scopus, WOS—web of science, PsycInfo and Google Scholar indexed databases were searched using the terms COVID-19, Coronavirus, mental health, anxiety, depression, and well-being crossed with perinatality-related terms (i.e., pregnancy, maternal mental health, maternal mental disorder, perinatal period). Following the need to accelerate the searches, as rapid reviews require, they were performed in the period from December 2020 to January 2021. The selection of material followed the reading of the titles and abstracts of identified publications. Articles were included if they fulfilled the following PICOS (population, intervention or exposure, comparison, outcomes, study design) eligibility criteria.

### 2.2. Population

Women who were pregnant at the time of the first wave of COVID-19 outbreak in their country.

### 2.3. Intervention/Exposure

Studies focusing on mental health outcomes (e.g., depression, anxiety, insomnia, post-traumatic stress disorder) in the target population during the COVID-19 pandemic.

### 2.4. Comparison

This is not applicable for the aim of this rapid review.

### 2.5. Outcomes

We looked at the following outcomes: psychological symptomatology (e.g., self-reported depression, anxiety, insomnia, post-traumatic stress disorder).

### 2.6. Study Design

We included studies with primary data collection.

### 2.7. Selection Criteria

The inclusion criteria were being published in English, reporting primary data and having the full-length text available, being original articles with at least 100 participants, being about the new coronavirus pandemic (COVID-19), and referring exclusively to its psychological consequences for women who were pregnant during the outbreak or were within the first year postpartum. The exclusion criteria were being editorials, letters or commentaries. We excluded articles that did not consider psychological aspects during pregnancy and abstracts without the full text available. A total of 116 articles were found in the initial search. After duplicates and papers without full texts available were removed, 41 full texts of possibly pertinent studies were assessed for eligibility and were independently screened by both authors to reduce the selection bias.

Finally, of a total of 116 publications found, 17 manuscripts met the aforementioned inclusion criteria; therefore, they were considered eligible and were included in the rapid review. Narrative synthesis was applied to analyze the relevant papers grouped under themes.

**Figure 1:**
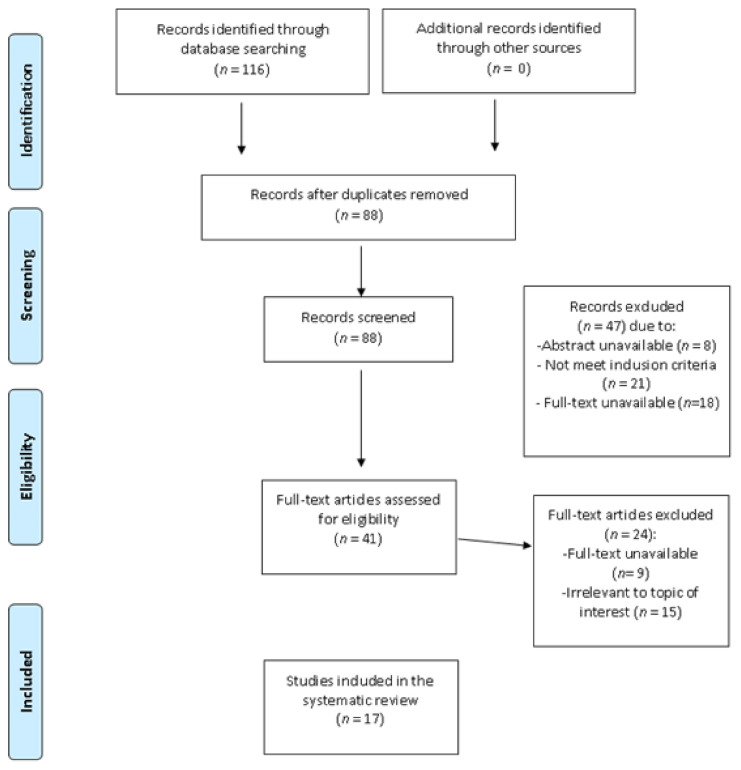
Prisma flowchart of information through the different phases of the review.

The study selection process is illustrated by the PRISMA flow chart shown in Figure 1.

### 2.8. Data Extraction

The study characteristics of the included papers were extracted by the two authors independently, and relevant information is shown in Table 1, including country, population, number of participants, study design, measurement tools and main results.

## 3. Results

### 3.1. Maternal Mental Health

All of the identified papers suggest that the COVID-19 pandemic can have a significant impact on maternal mental health, mainly in the form of anxiety and depressive symptoms. The prevalence of depression and anxiety in pregnant women has significantly increased since the spread of COVID-19 disease. Pregnant women during the COVID-19 pandemic reported more psychological symptomatology compared to pregnant women before the COVID-19 outbreak.

### 3.2. Countries

The studies included in the rapid review consider participants from China [22,23,24], Canada [18,25,26,27], Turkey [28,29], Argentina [3], Iran [30], Qatar [31], Spain [32], Italy [33,34], Pakistan [35], and Japan [36].

### 3.3. Participants

All the studies involved women who were at least 18 years old. Most of the papers concerned studies addressing women during pregnancy [3,18,22,23,25,26,33], two of which were case–control studies comparing pregnant and non-pregnant women [3,23]. Only one study [3] longitudinally monitored the population throughout the lockdown. In this case, participants were divided into two groups: 102 pregnant women and a control group of 102 non-pregnant women. One study was a case–control study [18] that considered pregnant women before the COVID-19 pandemic and pregnant women during the pandemic; finally, one contribution [27] considered pregnant and first-year postpartum women, assessed before and during the pandemic. A Chinese survey [22] compared the mental health status of pregnant women before the declaration of the COVID-19 epidemic and after. Only two studies [27,32] considered both pregnancy and the postpartum period.

### 3.4. Instruments

As regards the administered instruments, all studies adopted self-reports; seven studies delivered only one questionnaire, the rest multiple measures.

As concerns depression, seven studies applied the Edinburgh Postnatal Depression Scale (EPDS) [37], a 10-item self-report questionnaire addressing perinatal depressive symptoms within the last 7 days. The overall score is computed by adding items on a four-point Likert scale. Higher scores reflect more depressive symptoms.

Three others applied self-report depression symptoms scales, although these were not specific for pregnancy and the postpartum period: the Center for Epidemiological Studies Depression Scale [38]; the Beck Depression Inventory II [39]; the Patient Health Questionnaire 9 [40].

With respect to anxiety, only two studies evaluated perinatal anxiety: one study with a questionnaire including ten items specifically addressing feelings about the health of the baby and her/his birth [41], and the other administered the Cambridge Worry Scale (CWS) [42] to assess pregnancy-specific anxiety as well as general anxiety, whereas the majority of scholars applied generic anxiety questionnaires. Four administered the State–Trait Anxiety Inventory (STAI) [43]; one study applied the Generalized Anxiety Disorder Scale 7 (*G*AD**-7**) [44], one the Patient-Reported Outcomes Measurement Information System (PROMIS) Anxiety Adult seven-item short form [45], and one the Visual Analog Scale (VAS) for anxiety [46].

Some studies used a combined measure for depression and anxiety: two applied the Hospital Anxiety and Depression Scale (HADS) [47], and one the Patient Health Questionnaire Anxiety–Depression Scale (PHQ-ADS) [48].

One study administered the Depression Anxiety Stress Scales 21 (DASS 21) [49] to distinguish between the affective syndromes of depression, anxiety and tension/stress.

Three studies resorted to the the Positive and Negative Affect Schedule (PANAS) [50] to evaluate mood or emotion.

Global psychological distress was also measured through the Kessler Psychological Distress Scale (K10) [51] in two papers, and the Symptom Checklist—90 (SCL-90) [52] in one paper.

Finally, specific measures of other variables included in the contributions were evaluated, but none were specific to perinatality, with the exception of one study measuring the infants’ APGAR (Adaptability, Partnership, Growth, Affection, and Resolve)[53].

### 3.5. Prevalence of Depression and Anxiety Symptoms

The prevalence of depression and anxiety reported was similar for all studies considered. With regards to the prevalence of depression in Qatar, for example, 39.2% of pregnant women presented depressive symptomatology [31]; in Turkey, the prevalence was 56.3% [28]; in Iran, 32.7% of the participants had symptoms of depression [30]; 58% in Spain [32]; in Canada, the studies indicated values close to 40% (37% [25]; 40.7% [27]); in China, 29.6% of women in Wu’s study [22] and 33.71% among the 2883 women involved in Sun et al.’s survey [24] referred to symptoms of depression. Concerning anxiety symptoms, in Qatar, a 34.4% prevalence of anxiety was identified [31]; 51% has been reported in Spain [32]; in Canada rates from 56.6% [25] to 72% [27] were detected, which are close to the Italian prevalence of 68% [33], while two Turkish studies found rates of 64.5% [28] and 43.9% [30], respectively.

### 3.6. Comparison between Pre- and Post-COVID Depressive and Anxiety Symptoms

More specifically, one of the four studies from Canada (*n* =1987) found significantly higher anxiety and depressive symptoms compared to the scores in pregnant women before the COVID-19 pandemic, with 37% self-referring clinical levels of depression and 57% self-referring clinical levels of anxiety [25]. In Davenport et al.’s investigation [27], 900 women were involved: 58% were pregnant and 42% were in the first year postpartum. Pre-pandemic and current values were assessed for each group. It emerged that an EPDS score > 13 was self-reported in 15% pre-pandemic mothers and in 40.7% during the COVID 19. Moderate to high anxiety (STAI-state score > 40) was reported in 29% of women before the pandemic vs. 72% of women during its course.

In another Canadian survey, two cohorts of pregnant women were recruited, one prior to the COVID-19 pandemic (*n* = 496) and the other one (*n* = 1258) was enrolled online during the pandemic [18]. Researchers have shown that the latter reported more depressive and anxiety symptoms than pregnant women assessed before the COVID-19 pandemic. In addition, the COVID-19 women reported higher levels of dissociative symptoms and of post-traumatic stress disorder symptoms, and also described more negative affectivity and less positive affectivity than the pre-COVID-19 cohort did. In addition, this study showed that pregnant women assessed within the pandemic context with a previous psychiatric diagnosis or coming from a low-income background were more inclined to develop psychiatric symptoms.

The latter result contrasts with evidence from another study [31]: despite the main findings of Farrel’s research revealing that 34.4% of women reached clinical levels for anxiety and 39.2% for depression, these analyses did not reveal any association between these symptoms and previous mental health, occupation, pregnancy complications and gestational age. These results highlight that the worsening of psychiatric symptoms could be attributed to the psychological impact of the pandemic and to the containment measures. Similarly, Effati-Daryani et al. [30] showed that among their sample of 205 women, based on the scores obtained in DASS-21, 67.3% were in the normal range, 32.7% were identified to have symptoms of depression (12.7% mild, 10.7% moderate, 7.3% severe and 2.0% extremely), 56.1% were in the normal range, and 43.9% had symptoms of anxiety (17.6% mild, 12.2% moderate, 6.3% severe and 7.8% very severe). As emerged from Farrel’s aforementioned study, the evidence showed no statistically significant relationship between gestational age and depression, stress, and anxiety levels (*p* > 0.05).

A multi-center cross-sectional study [22] provided the opportunity to compare the mental health status of pregnant women before and after the declaration of the COVID-19 epidemic. Pregnant women assessed after the abovementioned declaration had significantly increased rates of depressive symptoms (26.0% vs. 29.6%) compared to women evaluated before the declaration. Additionally, the prevalence of depressive symptoms increased along with the increase in the number of newly confirmed cases, suspected infections and deaths. This evidence is consistent with Sun et al.’s study [24] that demonstrated that the prevalence of perinatal depression increased with the increasing number of confirmed cases of COVID-19 patients. In particular, among the 2883 women involved in the survey, 33.71% had depressive symptoms, 27.02% showed mild depression, 5.24% moderate depression, and 1.46% severe depression.

### 3.7. Comparison between Pregnant versus Non-Pregnant Women’s Mental Health

Regarding the prevalence of anxiety and depressive symptoms during the pandemic compared between pregnant and non-pregnant women, discordant results emerged from the two studies considered [3,23]. The first one [3] demonstrated that, during quarantine, both pregnant and non-pregnant women showed a gradual increment in psychopathological measures and a decline in positive affect. However, the group of pregnant women showed a more pronounced increase in depression, anxiety and negative affect than the non-pregnant women did. In addition, pregnant women showed a more evident decrease in positive affect. On the contrary, in the other study [23], pregnant women seemed to have an advantage when facing mental problems; really, they showed lower levels of depression, anxiety, insomnia and post-traumatic stress disorder (PTSD) than non-pregnant women. Specifically, 5.3%, 6.8%, 2.4%, 2.6%, and 0.9% of pregnant women, respectively, presented symptoms of depression, anxiety, physical discomfort, insomnia, and PTSD, whereas non-pregnant women’s prevalences were 17.5% (depression), 17.5% (anxiety), 2.5% (physical discomfort), 5.4% (insomnia), and 5.7% (PTSD).

Taken together, the data that emerged from the papers included in this review suggest that the COVID-19 outbreak had a moderate to severe effect on the mental health of pregnant women; actually, the prevalence of psychological symptoms (mainly depression and anxiety) has significantly increased with the diffusion of COVID-19.

### 3.8. Beyond Depression and Anxiety: Specific Maternal Worries and Fears

In addition to the common psychiatric symptoms of depression and anxiety, some of the included studies also reported a high prevalence of fear, which represents the most reported symptom in pregnant women [25,26,29,31,33,34,35,36].

The concerns regarding infection were mainly for the pregnancy and for their families and children. Many women in the reviewed studies from different countries expressed worries about their own health and that of their unborn children in relation to the pandemic [25,29,31,33,35,36], concerns about delivery (e.g., whether their partner will be present, giving birth) and the baby’s health (e.g., something being wrong with the infant) [26].

In particular, two studies reported evidence that pregnant women experience great anxiety regarding the fear of transmitting the virus vertically to their baby [29,33]. Saccone et al. [33] pointed out that almost half of the women (46%) had worries about transmitting the infection to their infants. In the survey headed by Akgor et al. [29], the authors found that 82.5% (*n* = 245) of the pregnant women involved in the research reported high anxiety regarding the vertical transmission of the disease to their babies during delivery if they were infected with COVID-19 [29].

Consistently with this, in another survey [31] on 552 mothers, 353 (64%) women were highly aware and worried about the COVID-19 pandemic (i.e., fears of carrying the virus, vertical transmission causing harm to fetuses, vulnerability). This finding emerged despite the fact that 64% of respondents did not acknowledge any impact of the COVID-19 pandemic on their mental well-being.

In a cross-sectional survey on 288 women accessing maternity services in Qatar, Farrel et al. [31] identified worries about pregnancy in 143 women and concerns about family and children in 189 of them. A high prevalence of fear of abnormal perinatal consequence was also detected; in one study conducted in Italy, over half of the mothers were worried that COVID-19 could cause a fetal structural anomaly, fetal growth restriction or preterm delivery [34]. Additionally, more than half of the pregnant women involved in another survey (66%, *n* = 196) were worried about pregnancy problems if their visits to the hospital were delayed or cancelled [29].

Furthermore, Lebel et al. [25] identified that the elevated depression and anxiety symptoms during the COVID-19 pandemic were significantly associated with COVID-19-related concerns, i.e., threats to their baby’s health and to their own lives, worries about not receiving enough care during pregnancy, and also worries due to social isolation. These levels are much higher than what is typical for pregnant women and those reported by the rest of the community during the COVID-19 pandemic [25].

### 3.9. Protective Factors

However, several of the reviewed studies also focused on some possible factors that may mediate/moderate the impact of the pandemic on women’s mental health. Some scholars reported that increased perceived social support and support effectiveness were associated with lower mental health symptoms, and appeared to be protective factors against depression and anxiety [25,26,27]. Similarly, a Japanese survey including 1777 pregnant women demonstrated that a lack of social support is significantly related to depressive symptoms [36].

These results are in line with previous literature that proved that better social support was related to decreased depression and anxiety symptoms during both pregnancy and postpartum [54,55]. As is known, life during a pandemic is characterized by isolation, social distancing, restrictive measures and the limitation of movement, all of which can lead women to experience a lack of social support from friends, relatives, and partners [56], with negative consequences for mental health, as mentioned above.

Physical activity has also been investigated in terms of its protective function for psychological symptoms. Specifically, four studies showed that physical activity is related to reduced mental health problems. Being involved in regular physical activity during the COVID-19 pandemic represents a protective factor for the onset of anxiety and depressive symptoms in pregnant [22,25,27,28] or postpartum women [27], as confirmed by Lebel et al. [25].

## 4. Discussion

The present rapid review was aimed at describing the current scientific evidence on the psychological impact of the COVID-19 outbreak on mother’s mental health in the perinatal period. We chose a rapid review with the aim of providing evidence in a “timely and cost-effective manner”, as stated by the WHO [21]. Indeed, perinatality is to be considered as a priority in the primary care system. An effective means of identification of the condition of women and their infants from pregnancy to the first year of life of the children may inform the management of potential mental health disorders, and guide efficacious preventive interventions. Although the current review may not be considered exhaustive, our findings confirm that the COVID-19 pandemic has a considerable impact on the psychological health of pregnant and postpartum women. Indeed, although lacking multicentered studies, research from different countries and cultures has shown an increased prevalence of depression and anxiety among mothers during COVID-19 compared to similar pre-COVID19 pandemic mothers [18,22,25,26,27,28,31,33,34]. Hence, an accurate screening approach should be implemented for women in the peripartum. This is especially true in the face of healthcare systems that are not able to respond to the progressive increase in the demand for services. Such a situation seems particularly relevant to healthcare systems under the pressure of the COVID-19 pandemic emergency, helping to reduce the workload by referring only the screened, most vulnerable women for targeted intervention.

It is noteworthy that most studies were carried out through web-based questionnaires. This modality seems particularly useful in the abovementioned low-resource contexts. Computerized screening should also be favored since it has been shown that people tend to reveal more personal information through the computer and feel a greater sense of anonymity, increasing the likelihood of participation [57]. Some studies also detected, in addition to depressive and anxiety symptoms, higher percentages of post-traumatic stress disorder, dissociation, and distress [18], and higher levels of negative affectivity [3,18]. Moreover, independently of identified psychological symptomatology, high levels of awareness and concerns about the COVID-19 pandemic emerged, especially fears of carrying the virus and vertical transmission causing harm to fetuses [25,29,31,33,35,36]. These are relevant issues in that maternal malaise is not limited to ordinary screened psychological problems (i.e., depression and anxiety). Traumatic responses and emotional dysregulation may also affect mothers and their infants after pregnancy, with relevant long-term psychophysiological effects [58,59,60,61]. Specific attention to these vulnerabilities must be considered in order to provide efficacious interventions.

## 5. Limitations

The findings of this review have to be seen in light of some limitations. First, grey literature was excluded, and the articles included were limited to those in the English language within the selected keywords and databases. For this reason, the review cannot claim to be representative of all studies addressing the topic under investigation; therefore, the evidence that emerged could be overestimated or underestimated.

Additionally, only two surveys compared mental health outcomes for pregnant women with non-pregnant women during pandemic, only one study compared pregnant women before the COVID-19 pandemic and pregnant women during the pandemic, and only one considered pregnant and first-year postpartum women assessed before and during the pandemic. The paucity of studies makes it difficult to point out the differences between being pregnant during a pandemic and in another period.

Moreover, no standardized quality appraisal of the included papers was carried out, as is usual in rapid evidence reviews [62]. This necessitates great caution in the interpretation of the review’s findings. Actually, the reviewed studies diverged with respect to enrollment modalities and the samples’ characteristics. Additionally, there was significant variability in the assessment measures that limits the generalizability of our findings; as well, in some cases, differences in the symptoms emerged, even though the same questionnaire was administered. Therefore, to improve the screening and prevention/intervention programs, a more rigorous study design is required, which should include the calculation of effect sizes, control groups, and a longitudinal perspective [14,63].

Essentially, every study resorted to self-report questionnaires. Even though self-report measures are commonly administered in studies addressing maternal psychological functioning in the perinatal period, biased responses may not be excluded. Furthermore, only a few studies included instruments specific to the pre- and postpartum period, which may cause misleading conclusions. Besides this, resorting to such types of instruments does not allow us to distinguish between transient maternal malaise and more structured psychopathology, which is important to intervention; that said, they make a crucial contribution in prevention programs.

Moreover, even though some of the reviewed studies considered additional variables (i.e., social support, physical activity) that may buffer the impact of the COVID-19 pandemic on mother’s psychological symptomatology [22,25,26,27,28,36], future studies should consider many of the risk factors that have been identified in the literature as relevant intervening variables, such as maternal SES and education, childbirth experiences, comorbidity, romantic couple adjustment, infant temperament, and breastfeeding [64,65,66,67,68]. From a research perspective, the interrelationship between these variables should be investigated through path analysis and linear structural relations modeling to understand their contributions to the outcomes for mothers and children.

## 6. Conclusions

The present review provides valuable clinical suggestions that should be carefully monitored during the evaluation of women during perinatality.

In fact, the COVID-19 pandemic adds numerous risk factors for the mental health of mothers during the perinatal period. Longitudinal, cohort, multicenter studies should be carried out in order to promote standardized screening and intervention guidelines to support pregnant and postpartum women during the COVID-19 outbreak, and to promote healthy family functioning. The identification of risks and protective factors during the current pandemic is particularly important, especially considering the long-term effect that maternal mental health has on a child’s development. Finally, despite the acknowledged distress linked to such a situation, it may offer the possibility to develop pioneering online methods to detect psychological problems and deliver early mental health interventions to mothers and their infants.

## Figures and Tables

**Table 1:** Studies of mental health concerns related to COVID-19 in pregnant and/or postpartum women, considering countries of recruitment, main characteristics of the sample, methodology and tools of measurement, and main results.

Authors and Publication Year	Country of Origin	Participants	Study Design	Study Instruments	Results
López-Morales, et al., 2020 [3]	Argentina	204 women divided into two groups: a pregnancy group with 102 pregnant women, and a control group with 102 non-pregnant women.	Longitudinal study	BDI-II; STAI; PANAS	The total sample showed a gradual increase in psychopathological indicators and a decrease in positive affect. Pregnant women reported a greater increase in depression, anxiety and negative affect than the control group.
Berthelot et al., 2020 [18]	Canada	*n* = 496 pregnant women before the COVID-19 pandemic; *n* = 1258 during the pandemic	Case–control study	K10, PCL-5, DES-II, PANAS	Pregnant women assessed during the pandemic reported more severe symptoms of depression and anxiety, higher levels of negative affectivity, lower levels of positive affectivity, and more symptoms of PTSD and dissociation than women from the pre-COVID-19 cohort.
Wu et al., 2020 [22]	China	4124 pregnant women (from 1 January 2020, to 9 February 2020); 2839 were assessed before the coronavirus epidemic was publicly declared and 1285 assessed after this time (After 20 January 2020)	Multi-center cross-sectional study	EPDS	Pregnant women assessed after the declaration of coronavirus disease had significantly higher rates of depressive symptoms than women assessed before the declaration (26.0% vs. 29.6%). The depressive rates were positively associated with the number of newly confirmed cases of coronavirus disease (*p* = 0.003), suspected infections (*p* = 0.004), and deaths per day (*p* = 0.001).
Zhou et al., 2020 [23]	China	859 participants: 544 pregnant women and 315 non-pregnant women	Cross-sectional study	PHQ-9, GAD-7, ISI, SCL-90, PCL-5	Pregnant women reported fewer depression, anxiety, insomnia and and post-traumatic stress disorder (PTSD) symptoms than non-pregnant women. The prevalence rates among pregnant women were 5.3% (depression), 6.8% (anxiety), 2.4% (physical discomfort), 2.6% (insomnia), 0.9% (PTSD). The prevalence among non-pregnant was 17.5% (depression), 17.5% (anxiety), 2.5% (physical discomfort), 5.4% (insomnia), 5.7% (PTSD)
Sun et al., 2020 [24]	China	2883 participants: prenatal women in the third trimester and postnatal women within 7 days after delivery	Cross-sectional study	EPDS; APGAR—family function scale	33.71% of the participants had depressive symptoms (27.02% mild depression, 5.24% moderate depression, 1.46% severe depression). The prevalence of perinatal depression increased along with the increasing number of confirmed cases of COVID-19.
Lebel, MacKinnon, 2020 [25]	Canada	1987 pregnant women	Cross-sectional study	EPDS; PROMIS Anxiety Adult 7-item short form; pregnancy-related anxiety questionnaire; SSEQ; ISEL; Godin–Shephard Leisure-Time Exercise Questionnaire	37.0% of respondents reported clinically elevated symptoms of depression, 46.3% had moderately elevated anxiety symptoms and 10.3% severely elevated anxiety symptoms. Regarding anxiety symptoms, 56.6% had clinically elevated anxiety symptoms and 67.6% had clinically elevated pregnancy-related anxiety. Measures of anxiety and depressive symptoms were moderately to strongly associated with each other, and negatively associated with perceived social support. Anxiety and depressive symptoms are significantly related to COVID-19-specific worries (e.g., effects on baby’s health) and social isolation.
Khoury et al., 2021 [26]	Canada	303 pregnant women	Cross-sectional study	CWS; CES-D ; ISI—Insomnia Severity Index MSPSS*;* Cognitive appraisal	57% of the sample reported clinically elevated depression, > 30% reported elevated worries, and 19% reported elevated insomnia. Depression and anxiety levels were higher than non-COVID pregnant samples. Social isolation, financial and relationship difficulties and risk of COVID-19 were associated with mental health outcomes. Higher social support exerts a protective function, particularly for those who appraise the impact of COVID-19 to be more negative.
Davenport et al., 2020 [27]	Canada	900 women: 520 (58%) were pregnant and 380 (42%) were in the first year after delivery; current and pre-pandemic values were assessed for each	Cross-sectional study	EPDS; STAI; Self-reported physical activity	An EPDS score > 13 was self-identified in 15% of the respondents pre-pandemic and in 40.7% currently. Moderate to high anxiety was identified in 29% of women before the pandemic and in 72% of women currently.
Sut & Kucukkaya, 2020 [28]	Turkey	403 pregnant women	Cross-sectional study	HADS	The prevalence of anxiety and depression in pregnant women during the COVID-19 pandemic was 64.5% and 56.3%, respectively, much higher than the reported pre-pandemic prevalence.
Akgor et al., 2021 [29]	Turkey	297 pregnant women	Prospective study	HADS	60.3% of pregnant women thought COVID infection risk was higher in their babies compared to themselves, and 82.5% had concerns about transmitting the infection to their babies during delivery if they became infected with COVID-19; 79.5% were afraid of getting a COVID infection from the hospital during their follow-up or the birth; 51.5%, were concerned about not being able to carry out regular antenatal care and 66% were concerned about pregnancy complications if their follow-ups were postponed or cancelled. The fear of infection of the fetus revealed elderly age and having anxiety as the unique significant risk factors.
Effati-Daryani et al., 2020 [30]	Iran	205 pregnant women	Cross-sectional study	DASS-21	67.3% of women had normal status and 32.7% had symptoms of depression. Regarding stress, 67.3% of participants showed normal levels and 32.7% of them had symptoms of stress. In the anxiety test, 43.9% had symptoms of anxiety. As for the pregnancy trimester, no statistically significant associations between depression, stress and anxiety were found (*p* > 0.05).
Farrell et al., 2020 [31]	Qatar	288 women	Cross-sectional study	PHQ-ADS	The survey results revealed a high prevalence of anxiety and depressive symptomatology (34.4 and 39.2%, respectively). These rates appeared much higher than the reported pre-pandemic prevalence.
Chaves, 2021 [32]	Spain	724 women (450 pregnancy, 274 postpartum) in antenatal period or who had given birth in the previous six months at the time of the study and during the initial time of the COVID-19 emrgency state in Spain	Cross-sectional study	EPDS; PANAS; SWLS	58% of women reported depressive symptoms, assessed as EPDS > 11; 51% of women reported anxiety symptoms.
Saccone et al., 2020 [33]	Italy	100 women were enrolled (17 in the first trimester of pregnancy, 35 in the second, and 48 in the third)	Cross-sectional study	STAI; IES-R; VAS	COVID-19 outbreak had a moderate to severe impact on pregnant women’s mental health; 53% of participants rated the psychological impact as severe; 46% reported high anxiety with respect to the vertical transmission of the disease. The psychological impact of COVID-19 pandemic was more severe in women in the first trimester of pregnancy. They reported significantly higher mean STAI scores, higher rates of STAYscore > 36, higher mean scores at VAS for anxiety for COVID-19 vertical transmission, and higher rates of VAS score.
Mappa et al., 2020 [34]	Italy	178 pregnant women	Prospective observational study	STAI-T, STAI-S	In total, 77% of pregnant women experienced a greater psychological impact as well as higher anxiety during the COVID-19 outbreak; 75% of pregnant women reported a fear of going to the hospital. About maternal concerns of the effect of infection: 37% were concerned about not having enough information about the effects of COVID-19 on pregnancy, 41% about not being able to carry out regular antenatal care and 22% that they had come into contact with the virus. Fear that COVID-19 could induce fetal structural anomalies was present in 46.6%, fear of fetal growth restriction in 65.2% and fear of preterm birth in 51.1% of women.
Shahid et al., 2020 [35]	Pakistan	552 pregnant women	Descriptive cross-sectional study	Kessler-10 scale (K-10), EPDS	In total, 64% of women experienced no effect on their mental health, while 36% declared that the COVID-19 pandemic had a big impact on their mental health; 27.3% of pregnant women revealed mild signs of psychological effects, 7.2% had moderate signs and 1.5% of participants had severe signs of psychological impact. Concerning the prevalence of depression and anxiety, 61% of pregnant women neither felt depressed nor anxious; 39% declared that the COVID-19 pandemic had caused them depression and anxiety, while 33% (182 women) were found to have possible depression, and 6% scored 30 on the EPDS, indicating maximum depression.
Matsushima & Horiguchi, 2020 [36]	Japan	1777 pregnant women	Cross-sectional study	EPDS	A high percentage of pregnant women were found to have depressive symptoms. It also emerged that COVID-19-related variables (i.e., perceived risk for infection, fear of decreasing economic wealth and social support) were significantly associated with depressive symptoms.

APGAR = family function scale; BDI II = Beck Depression Inventory II; CWS = Cambridge Worry Scale; CES-D = Center for Epidemiologic Studies Depression Scale; DASS-21 = Depression Anxiety Stress Scales 21; DES-II = Dissociative Experiences Scale; EPDS = Edinburgh Postnatal Depression Scale; GAD-7 = Generalized Anxiety Disorder Scale 7; HADS = Hospital Anxiety and Depression Scale; IES-R = Impact of Event Scale-Revised; ISEL = Interpersonal Support Evaluation list; ISI = Insomnia Severity Index; K10 = 10-item Kessler Psychological Distress Scale; MSPSS = Multidimensional Scale of Perceived Social Support; PANAS = Positive and Negative Affect Schedule; PCL-5 = Post-Traumatic Checklist for DSM-5; PHQ-9 = Patient Health Questionnaire; PHQ-ADS = Patient Health Questionnaire Anxiety Depression Scale; PROMIS (Patient-Reported Outcomes Measurement Information System) Anxiety Adult 7-item short form; Symptom Checklist-90 (SCL-90); STAI = State–Trait Anxiety Inventory; SSEQ = Social Support Effectiveness Questionnaire; SWLS = Satisfaction With Life Scale; VAS = Visual Analog Scale for anxiety.

## Data Availability

Not applicable.

## References

[1] Brooks S.K., Webster R.K., Smith L.E., Woodland L., Wessely S., Greenberg N., Rubin G.J. (2020). The psychological impact of quarantine and how to reduce it: Rapid review of the evidence. Lancet.

[2] Torales J., O’Higgins M., Castaldelli-Maia J.M., Ventriglio A. (2020). The outbreak of COVID-19 coronavirus and its impact on global mental health. Int. J. Soc. Psychiatry.

[3] López-Morales H., Del Valle M.V., Canet-Juric L., Andrés M.L., Galli J.I., Poó F., Urquijo S. (2020). Mental health of pregnant women during the COVID-19 pandemic: A longitudinal study. Psychiatry Res..

[4] Spinola O., Liotti M., Speranza A.M., Tambelli R. (2020). Effects of COVID-19 epidemic lockdown on postpartum depressive symptoms in a sample of Italian mothers. Front. Psychiatry.

[5] Kajdy A., Feduniw S., Ajdacka U., Modzelewski J., Baranowska B., Sys D., Pokropek A., Pawlicka P., Kaźmierczak M., Rabijewski M. (2020). Risk factors for anxiety and depression among pregnant women during the COVID-19 pandemic: A web-based cross-sectional survey. Medicine.

[6] American Psychiatric Association (2013). Diagnostic and Statistical Manual of Mental Disorders (DSM-5®).

[7] Darvill R., Skirton H., Farrand P. (2010). Psychological factors that impact on women’s experiences of first-time motherhood: A qualitative study of the transition. Midwifery.

[8] George A., Luz R.F., De Tychey C., Thilly N., Spitz E. (2013). Anxiety symptoms and coping strategies in the perinatal period. BMC Pregnancy Childbirth.

[9] Bener A., Gerber L.M., Sheikh J. (2012). Prevalence of psychiatric disorders and associated risk factors in women during their postpartum period: A major public health problem and global comparison. Int. J. Women’s Health.

[10] Dennis C.L., Falah-Hassani K., Shiri R. (2017). Prevalence of antenatal and postnatal anxiety: Systematic review and meta-analysis. Br. J. Psychiatry.

[11] Harville E.W., Xiong X., Buekens P. (2010). Disasters and perinatal health: A systematic review. Obstet. Gynecol. Surv..

[12] O’Connor E., Senger C.A., Henninger M.L., Coppola E., Gaynes B.N. (2019). Interventions to prevent perinatal depression: Evidence report and systematic review for the US Preventive Services Task Force. JAMA.

[13] Brooks S.K., Weston D., Greenberg N. (2020). Psychological impact of infectious disease outbreaks on pregnant women: Rapid evidence review. Public Health.

[14] do Amaral W.N., de Moraes C.L., Dos Santos Rodrigues A.P., Noll M., Arruda J.T., Mendonça C.R. (2020). Coronavirus Infections and Neonates Born to Mothers with SARS-CoV-2: A Systematic Review. Healthcare.

[15] Di Giorgio E., Di Riso D., Mioni G., Cellini N. (2020). The interplay between mothers’ and children behavioral and psychological factors during COVID-19: An Italian study. Eur. Child Adolesc. Psychiatry.

[16] Wang Y., Chen L., Wu T., Shi H., Li Q., Jiang H., Zheng D., Wang X., Wei Y., Zhao Y. (2020). Impact of Covid-19 in pregnancy on mother’s psychological status and infant’s neurobehavioral development: A longitudinal cohort study in China. BMC Med..

[17] Naurin E., Markstedt E., Stolle D., Enström D., Wallin A., Andreasson I., Attebo B., Eriksson O., Martinsson K., Elden H. (2021). Pregnant under the pressure of a pandemic: A large-scale longitudinal survey before and during the COVID-19 outbreak. Eur. J. Public Health.

[18] Berthelot N., Lemieux R., Garon-Bissonnette J., Drouin-Maziade C., Martel É., Maziade M. (2020). Uptrend in distress and psychiatric symptomatology in pregnant women during the COVID-19 pandemic. Acta Obstet. Gynecol..

[19] Rasmussen S.A., Smulian J.C., Lednicky J.A., Wen T.S., Jamieson D.J. (2020). Coronavirus disease 2019 (COVID-19) and pregnancy: What obstetricians need to know. Am. J. Obstet. Gynecol..

[20] Meaney M.J. (2018). Perinatal maternal depressive symptoms as an issue for population health. Am. J. Psychiatry.

[21] Tricco A.C., Langlois E., Straus S.E., World Health Organization (2017). Rapid Reviews to Strengthen Health Policy and Systems: A Practical Guide.

[22] Wu Y., Zhang C., Liu H., Duan C., Li C., Fan J., Li H., Chen L., Xu H., Li X. (2020). Perinatal depressive and anxiety symptoms of pregnant women during the coronavirus disease 2019 outbreak in China. Am. J. Obstet. Gynecol..

[23] Zhou Y., Shi H., Liu Z., Peng S., Wang R., Qi L., Li Z., Yang J., Ren Y., Song X. (2020). The prevalence of psychiatric symptoms of pregnant and non-pregnant women during the COVID-19 epidemic. Transl. Psychiatry.

[24] Sun G., Wang F., Cheng Y. (2020). Perinatal depression during the COVID-19 epidemic in Wuhan, China. China.

[25] Lebel C., MacKinnon A., Bagshawe M., Tomfohr-Madsen L., Giesbrecht G. (2020). Elevated depression and anxiety symptoms among pregnant individuals during the COVID-19 pandemic. J. Affect. Disord..

[26] Khoury J.E., Atkinson L., Bennett T., Jack S.M., Gonzalez A. (2021). COVID-19 and mental health during pregnancy: The importance of cognitive appraisal and social support. J. Affect. Disord..

[27] Davenport M.H., Meyer S., Meah V.L., Strynadka M.C., Khurana R. (2020). Moms are not ok: COVID-19 and maternal mental health. Front. Glob. Women’s Health.

[28] Sut H.K., Kucukkaya B. (2020). Anxiety, depression, and related factors in pregnant women during the COVID-19 pandemic in Turkey: A web-based cross-sectional study. Perspect. Psychiatr. Care.

[29] Akgor U., Fadıloglu E., Soyak B., Unal C., Cagan M., Temiz B.E., Ak S., Gultekin M., Ozyuncu O. (2021). Anxiety, depression and concerns of pregnant women during the COVID-19 pandemic. Arch. Gynecol. Obstet..

[30] Effati-Daryani F., Zarei S., Mohammadi A., Hemmati E., Yngyknd S.G., Mirghafourvand M. (2020). Depression, stress, anxiety and their predictors in Iranian pregnant women during the outbreak of COVID-19. BMC Psychol..

[31] Farrell T., Reagu S., Mohan S., Elmidany R., Qaddoura F., Ahmed E.E., Corbett G., Lindow S., Abuyaqoub S.M., Alabdulla M.A. (2020). The impact of the COVID-19 pandemic on the perinatal mental health of women. J. Perinat. Med..

[32] Chaves C., Marchena C., Palacios B., Salgado A., Duque A. (2021). Effects of the COVID-19 pandemic on perinatal mental health in Spain: Positive and negative outcomes. Women Birth.

[33] Saccone G., Florio A., Aiello F., Venturella R., De Angelis M.C., Locci M., Bifulco G., Zullo F., Sardo A.D.S. (2020). Psychological impact of coronavirus disease 2019 in pregnant women. Am. J. Obstet. Gynecol..

[34] Mappa I., Distefano F.A., Rizzo G. (2020). Effects of coronavirus 19 pandemic on maternal anxiety during pregnancy: A prospectic observational study. J. Perinat. Med..

[35] Shahid A., Javed A., Rehman S., Tariq R., Ikram M., Suhail M. (2020). Evaluation of psychological impact, depression, and anxiety among pregnant women during the COVID-19 pandemic in Lahore, Pakistan. Int. J. Gynecol. Obstet..

[36] Matsushima M., Horiguchi H. (2020). The COVID-19 pandemic and mental well-being of pregnant women in Japan: Need for economic and social policy interventions. Disaster Med. Public Health Prep..

[37] Cox J.L., Holden J.M., Sagovsky R. (1987). Edinburghpostnataldepression scale (EPDS). Br. J. Psychiatry.

[38] Radloff L.S. (1977). The CES-D scale: A self report depression scale for research in the general population. Appl. Psychol. Meas..

[39] Beck A.T., Steer R.A., Brown G. (1996). Beck Depression Inventory–II.

[40] Kroenke K., Spitzer R.L., Williams J.B.W. (2001). The Patient Health Questionnaire (PHQ-9)–overview. J. Gen. Intern. Med..

[41] Rini C.K., Dunkel-Schetter C., Wadhwa P.D., Sandman C.A. (1999). Psychological adaptation and birth outcomes: The role of personal resources, stress, and sociocultural context in pregnancy. Health Psychol..

[42] Statham H., Green J.M., Kafetsios K. (1997). Who worries that something might be wrong with the baby? A prospective study of 1072 pregnant women. Birth.

[43] Spielberger C.D., Gorsuch R.L., Lushene R., Vagg P.R., Jacobs G.A. (1983). Manual for the State-Trait Anxiety Inventory.

[44] Spitzer R.L., Kroenke K., Williams J.B., Löwe B. (2006). A brief measure for assessing generalized anxiety disorder: The GAD-7. Arch. Intern. Med..

[45] Cella D., Riley W., Stone A., Rothrock N., Reeve B., Yount S., Amtmann D., Bode R., Buysse D., Choi S. (2010). The Patient-Reported Outcomes Measurement Information System (PROMIS) developed and tested its first wave of adult self-reported health outcome item banks: 2005–2008. J. Clin. Epidemiol..

[46] Hornblow A.R., Kidson M.A. (1976). The visual analogue scale for anxiety: A validation study. Aust. N. Z. J. Psychiatry.

[47] Zigmond A.S., Snaith R.P. (1983). The hospital anxiety and depression scale. Acta Psychiatr..

[48] Kroenke K., Wu J., Yu Z., Bair M.J., Kean J., Stump T., Monahan P.O. (2016). The patient health questionnaire anxiety and depression scale (PHQ-ADS): Initial validation in three clinical trials. Psychosom. Med..

[49] Lovibond S.H., Lovibond P.F. (1998). Manual for the Depression Anxiety Stress Scales.

[50] Watson D., Clark L.A., Tellegen A. (1988). Development and validation of brief measures of positive and negative affect: The PANAS scales. J. Personal. Soc. Psychol..

[51] Kessler R.C., Andrews G., Colpe L.J., Hiripi E., Mroczek D.K., Normand S.L., Walters E.E., Zaslavsky A.M. (2002). Short screening scales to monitor population prevalences and trends in non-specific psychological distress. Psychol. Med..

[52] Derogatis L.R. (1983). Manual for the SCL-90-R.

[53] Smilkstein G. (1978). The family APGAR: A proposal for a family function test and its use by physicians. J. Fam. Pract..

[54] Negron R., Martin A., Almog M., Balbierz A., Howell E.A. (2013). Social support during the postpartum period: Mothers’ views on needs, expectations, and mobilization of support. Matern. Child Health J..

[55] Gjerdingen D.K., Froberg D.G., Fontaine P. (1991). The effects of social support on women’s health during pregnancy, labor and delivery, and the postpartum period. Fam. Med..

[56] Caparros-Gonzalez R.A., Ganho-Ávila A., Torre-Luque A.D.L. (2020). The COVID-19 Pandemic Can Impact Perinatal Mental Health and the Health of the Offspring. Behav. Sci..

[57] Kingston D., Austin M.P., van Zanten S.V., Harvalik P., Giallo R., McDonald S.D., MacQueen G., Vermeyden L., Lasiuk G., Biringer A. (2017). Pregnant women’s views on the feasibility and acceptability of web-based mental health e-screening versus paper-based screening: A randomized controlled trial. J. Med Internet Res..

[58] Youssef n.A., Lockwood L., Su S., Hao G., Rutten B.P. (2018). The effects of trauma, with or without PTSD, on the transgenerational DNA methylation alterations in human offsprings. Brain Sci..

[59] Deuschle M., Hendlmeier F., Witt S., Rietschel M., Gilles M., Sánchez-Guijo A., Fañanas L., Hentze S., Wudy S.A., Hellweg R. (2018). Cortisol, cortisone, and BDNF in amniotic fluid in the second trimester of pregnancy: Effect of early life and current maternal stress and socioeconomic status. Dev. Psychopathol..

[60] Bosquet Enlow M., Devick K.L., Brunst K.J., Lipton L.R., Coull B.A., Wright R.J. (2017). Maternal lifetime trauma exposure, prenatal cortisol, and infant negative affectivity. Infancy.

[61] Olsen J.M. (2018). Integrative review of pregnancy health risks and outcomes associated with adverse childhood experiences. J. Obstet. Gynecol. Neonatal Nurs..

[62] Haby M.M., Chapman E., Clark R., Barreto J., Reveiz L., Lavis J.N. (2016). What are the best methodologies for rapid reviews of the research evidence for evidence-informed decision making in health policy and practice: A rapid review. Health Res. Policy Syst..

[63] Tahara M., Mashizume Y., Takahashi K. (2021). Coping Mechanisms: Exploring Strategies Utilized by Japanese Healthcare Workers to Reduce Stress and Improve Mental Health during the COVID-19 Pandemic. Int. J. Environ. Res. Public Health.

[64] Field T. (2018). Postnatal anxiety prevalence, predictors and effects on development: A narrative review. Infant Behav. Dev..

[65] Molgora S., Fenaroli V., Saita E. (2020). The association between childbirth experience and mother’s parenting stress: The mediating role of anxiety and depressive symptoms. Women Health.

[66] Rollè L., Prino L.E., Sechi C., Vismara L., Neri E., Polizzi C., Trovato A., Volpi B., Molgora S., Fenaroli V. (2017). Parenting stress, mental health, dyadic adjustment: A structural equation model. Front. Psychol..

[67] Vismara L., Sechi C., Neri M., Paoletti A., Lucarelli L. (2020). Maternal perinatal depression, anxiety, fear of birth, and perception of infants’ negative affectivity at three months. J. Reprod. Infant Psychol..

[68] Yang N., Che S., Zhang J., Wang X., Tang Y., Wang J., Huang L., Wang C., Zhang H., Baskota M. (2020). Breastfeeding of infants born to mothers with COVID-19: A rapid review. Ann. Transl. Med..

